# Three Distinct Roles for Notch in *Drosophila* R7 Photoreceptor Specification

**DOI:** 10.1371/journal.pbio.1001132

**Published:** 2011-08-23

**Authors:** Andrew Tomlinson, Yannis Emmanuel Mavromatakis, Gary Struhl

**Affiliations:** 1 Department of Genetics and Development, College of Physicians and Surgeons, Columbia University, New York, New York, United States of America; 2 Howard Hughes Medical Institute, College of Physicians and Surgeons, Columbia University, New York, New York, United States of America; New York University, United States of America

## Abstract

Receptor tyrosine kinases (RTKs) and Notch (N) proteins are different types of transmembrane receptors that transduce extracellular signals and control cell fate. Here we examine cell fate specification in the *Drosophila* retina and ask how N acts together with the RTKs Sevenless (Sev) and the EGF receptor (DER) to specify the R7 photoreceptor. The retina is composed of many hundred ommatidia, each of which grows by recruiting surrounding, undifferentiated cells and directing them to particular fates. The R7 photoreceptor derives from a cohort of three cells that are incorporated together following specification of the R2-R5 and R8 photoreceptors. Two cells of the cohort are specified as the R1/6 photoreceptor type by DER activation. These cells then activate N in the third cell (the R7 precursor). By manipulation of N and RTK signaling in diverse combinations we establish three roles for N in specifying the R7 fate. The first role is to impose a block to photoreceptor differentiation; a block that DER activation cannot overcome. The second role, paradoxically, is to negate the first; Notch activation up-regulates Sev expression, enabling the presumptive R7 cell to receive an RTK signal from R8 that can override the block. The third role is to specify the cell as an R7 rather than an R1/6 once RTK signaling has specified the cells as a photoreceptor. We speculate why N acts both to block and to facilitate photoreceptor differentiation, and provide a model for how N and RTK signaling act combinatorially to specify the R1/6 and R7 photoreceptors as well as the surrounding non-neuronal cone cells.

## Abstract 2

During specification of the R7 photoreceptor in the <I>Drosophila</I> eye, activation of Notch signaling leads to multiple responses within the cell, including antagonistic ones.

## Author Summary

Cells are often directed to their developmental fates by the signals they receive from other cells. The Drosophila eye has become a classic paradigm for studying such signaling, and in this system direct neighbor-to-neighbor signaling plays a large role. The R7 photoreceptor is directed to its fate by signals derived from two different neighboring cell types. One sends a signal that activates tyrosine kinase signaling in the R7 precursor, whereas the other activates the Notch signaling pathway. Here we examine Notch signaling and find that it induces three responses in the R7 precursor. We show that one role acts to inhibit the specification of the cell as a photoreceptor, while another role opposes this function, and acts to direct the cell to the photoreceptor fate. The third role specifies the cell as the specialized R7 photoreceptor rather than as the generic photoreceptor type. These results demonstrate that activation of a single signaling pathway can result in multiple cellular responses, even antagonistic ones.

## Introduction

During development, cells receive signals from local or distant sources and respond by sending other such signals. As a result, complex systems of spatial and temporal information emerge and individual cells make decisions about their fates based on this information. Remarkably, only a few ligands and receptors mediate these signaling events. Hence, the question arises as to how a small number of signaling pathways can generate elaborate cell patterns such as the array of photoreceptors, lens and pigment cells that make up each facet of the insect compound eye.

Two concepts have emerged. The first is the iterative use of signals. In this case, each signaling event alters the state of a cell and determines how it responds to subsequent signals. Accordingly, a cell can receive a sequence of signals over time and progress in a stepwise manner towards its ultimate fate. The second concept is the combinatorial use of the signals. In this case, the informative value of a signal depends on whether it is received alone or together with one or more additional signals. Both mechanisms appear to apply in most developmental contexts, but how they are used, either separately or in conjunction to dictate any given cell fate is poorly understood. Here we investigate how receptor tyrosine kinase (RTK) and Notch (N) signaling are used to specify a unique cell type: the R7 photoreceptor of the *Drosophila* eye.

**Figure 1:**
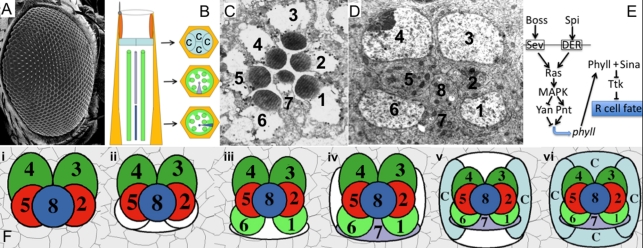
Details of the developing and adult ommatidia. (A) Scanning EM of the adult eye. Each facet corresponds to a single ommatidium. (B) A schematic adult ommatidium. The four cone cells (c, light blue) overlie the eight photoreceptors. Green labels the outer photoreceptors (R1-R6); R7 is colored purple and R8 is dark blue. R7 lies higher in the retina than R8. (C) TEM section through an ommatidium corresponding to the middle cross-section in (B). R7 is evident at this level. (D) TEM section through a developing ommatidium. At this stage the cone cells are yet to join the cluster. Presumptive photoreceptors are numbered. Note that R8 is the central cell at this stage. (E) Schematic summary of the RTK signaling that promotes the photoreceptor fate. Ras activation via Sev or DER signaling leads to the expression of Phyll, which targets Ttk for degradation. (F) Schematic summary of the incorporation and differentiation of the first seven cells added to the precluster. (i) The precluster is surrounded by many cells. (ii) Cells join the cluster at precise positions; the first three join on the R2/5/8 face. (iii) The two that contact R2/5 begin to differentiate as R1/R6. (iv) The cell between them waits and then subsequently begins differentiation as R7. Two cone cell precursors join the cluster in flanking positions. (v) Two more cone cells join to complete the four cone cell grouping that surrounds the photoreceptors. (vi) The 12 cell cluster with 8 photoreceptors and four cone cells is formed.

The *Drosophila* ommatidium is a complex assembly of 20 cells arrayed in a stereotypical pattern in which each cell can be uniquely identified by both its type and its position in the structure (Figure 1A–1C). Each ommatidium is composed of a core assembly of eight photoreceptors and four cone cells (non-neural lens elements) surrounded by an array of primary, secondary and tertiary pigment cells, and mechanosensory bristle complexes (Figure 1B) [1]. The core assembly arises in two distinct phases. In the first phase, a “precluster” of five cells withdraws from the cell cycle and differentiates into five photoreceptors in a stepwise fashion: first R8, then the pair R2/R5 and then the R3/R4 pair. In the second phase, the assembly grows by successive rounds of accretion of surrounding cells (Figure 1F). Cells are recruited to specific positions (niches; white cells in Figure 1F) on the surface of the growing cluster, and as they begin to differentiate they then contribute to the formation of new niches into which the next round of cells will be recruited [2],[3]. During the first round of accretion, a group of three undifferentiated cells is added to the R2/8/5 face of the precluster (Figure 1Fii). Upon joining, the two end cells, which abut the R2 and R5 cells, rapidly differentiate as the R1 and R6 cells (Figure 1Fiii). Several hours later, the middle cell, which abuts R8, differentiates as an R7 cell. The next rounds of accretion add cells first to the anterior and posterior faces of the unit (Figure 1Fiv), and then to the polar and equatorial faces (Figure 1Fv). Upon joining, these cells differentiate as non-neuronal cone cells. Thus, three distinct cell types are sequentially specified: first R1/6, then R7 and finally the cone cells (Figure 1Fvi) [2],[3].

Receptor tyrosine kinase (RTK) signaling is critically required for the specification of the R1-R7 photoreceptors. Acting through the Ras/MAPK pathway, it activates the transcription of *phyllopod* (*phyll*) [4],[5] that encodes an adapter protein that directs the ubiquitin ligase Sina to target the transcription factor Tramtrack (Ttk) for degradation (Figure 1E) [6]–[8]. Ttk is the major inhibitor of photoreceptor development: those cells that degrade it proceed to become photoreceptors, while those that do not become the other components of the ommatidium. RTK signaling is also involved in other aspects of ommatidial development, both before and after the choice to degrade Ttk is made. For example, moderate levels of RTK signaling regulate the survival and proliferation of cells before they are recruited to the cluster [9]–[11] as well as the differentiation of cone cells after the choice not to degrade Ttk is made [12]. In this article we focus exclusively on the RTK signaling events that determine whether a cell becomes a photoreceptor (Ttk degraded) or not (Ttk not degraded).

Two RTKs are required for specification of the R1/6/7 photoreceptors: the Drosophila EGF-Receptor (DER) and Sevenless (Sev). DER is ubiquitously expressed and is activated by its ligand Spitz, a diffusible peptide that is secreted by differentiating cells of the precluster and is required for the specification of all the six outer photoreceptors (R1–R6) [13],[14]. Sev is expressed in a complex manner; it is found in R3/4 and the mystery cells in the precluster, but at the time around R7 specification it is expressed at high levels in the R7 and cone cell precursors, and at low levels in the R1/6 progenitors. It is activated by a membrane bound ligand, Bride of Sevenless (Boss) presented on the surface of the R8 cell [15]–[18]. The R1/6/7 precursors contact R8 but the cone cell progenitors do not (Figure 1Fvi).

For many years only the RTK signaling pathway was clearly defined in the developing ommatidia. DER and Sev were seen as performing the same role in the R1/6 and R7 precursors respectively [14]. That is, they activated the Ras/MAPK pathway to degrade Ttk and induce photoreceptor differentiation (Figure 1E). However, the R1/6 and R7 cells are morphologically and physiologically distinct photoreceptor types, and although the activity of DER and Sev explains how they become photoreceptors, it does not explain what makes them different kinds of photoreceptors. Three models have been invoked to explain what differentiates the R1/6 and R7 photoreceptors. One model is that the timing or intensity of the RTK signal is the critical determinant. For example, a “clock” in the cells might meter the time of receipt of the RTK signal, and those receiving it early would become R1/6 types and those late would become R7s [14]. A second model sees Sev conferring a more potent activation of the Ras/MAPK pathway than DER, sufficient to engage additional outputs that select the R7 as opposed to the R1/6 fate. The third model posits that R1/6 cells, or perhaps the R8, express a second signal that acts in combination with Boss to direct the R7 precursors to their unique fates [3]. This third model was substantiated by the subsequent discovery that N signaling was required for specification of R7. Signaling by the N ligand Delta (Dl) on R1/6 activates N in the R7 precursor, directing it to the R7 fate [19],[20]. In the absence of Sev, the R7 precursor fails to differentiate as a photoreceptor and adopts the non-neuronal cone cell fate [17], and in the absence of the N signal it differentiates as an R1/6 photoreceptor [19],[20].

Thus, specification of the R7 fate requires the activation of two signaling pathways, providing a paradigm for studying how a cell “decodes” two signals to choose a particular fate. The role of the RTK signal in this process is relatively well understood but that of N has remained less clear. Here we have examined the roles played by N and find that it has (at least) three distinct roles in dictating R7 specification. First, N activity in the R7 precursor creates a barrier to RTK-induced photoreceptor differentiation, one that DER activation is not able to overcome. Second, it promotes Sev expression, providing the R7 precursor with a means to receive an additional RTK signal that overcomes the N-induced barrier to photoreceptor differentiation. Third, it provides an input that dictates the choice of R7 as opposed to R1/6 photoreceptor type. Extrapolating from these results, we propose a combinatorial model for the specification of the R1/6, R7 and cone cell fates by RTK and N signaling.

## Results

We define three distinct roles for N in R7 specification. A role in activating *sev* transcription was uncovered first; the remaining roles emerged from experiments in which this role was bypassed. Accordingly, we begin with the evidence that *sev* transcription is N dependent.

### Role 1—N Activity Up-Regulates *sev* Transcription to Specify the R7 Precursor as a Photoreceptor Rather Than a Cone Cell

#### (i) N activity regulates *sev* transcription

Sev is expressed in a complex pattern in the developing ommatidium; it is absent from R8, R2 and R5, barely detectable in R1 and R6, and accumulates highly in the remaining photoreceptors (R3, R4 and R7) as well as the cone cells [21]. Using a *sev* promoter fragment that reproduces the expression pattern of the native *sev* gene [22], we previously drove a constitutively activated form of N (sev.N^*^) and observed the cell-autonomous differentiation of R1 and R6 precursors as ectopic R7 photoreceptors [20]. This finding was one of several that established a causal relationship between N activity and the specification of the R7 versus the R1/6 fate [19],[20]. However, it posed the question of why sev.N^*^ activity is effective at transforming R1/6 precursors to R7s given that the *sev* promoter is normally expressed at such a low level in these cells. One simple explanation is that *sev* transcription is itself up-regulated by N activity. Accordingly, even an initially low level of sev.N^*^ expression in the R1/6 precursors might be expected to stimulate *sev* promoter activity, creating a positive auto-regulatory loop that amplifies expression of both the sev.N^*^ transgene as well as endogenous *sev*.

**Figure 2:**
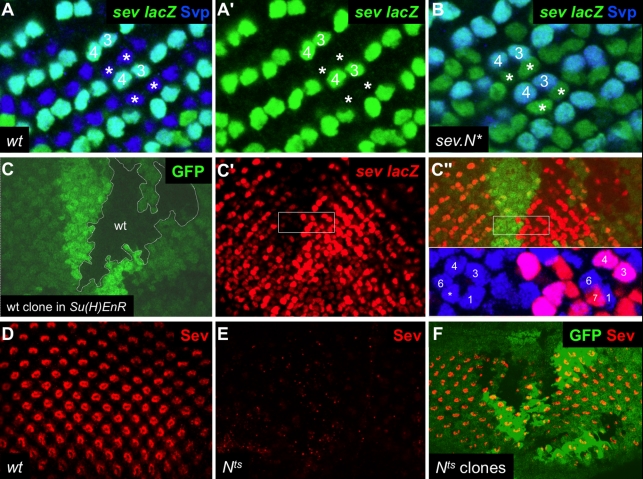
N regulates sev transcription. (A, B) Activated N induces up-regulation of *sev.lacZ*. (A) shows a wild type eye disc stained for *sev.lacZ* (green) and α-Svp (blue). The R1/6 precursors (asterisks) are labeled with α-Svp. (A') shows the same disc labeled for *sev.lacZ* alone. The asterisks mark the R1/6 precursors which do not express detectable levels of *sev.lacZ*. (B) Image of a sev.N^*^ eye disc stained as in (A). Here the cells in the R1/6 positions (asterisks) can be seen expressing high levels of β-Galactosidase (green). They do not express Svp because they differentiate as R7s. (C) Down-regulation of N signaling reduces *sev.lacZ* expression. Wild type clones (black) induced in a *sev.Su(H)EnR* background (green). The twin spots with two copies of *sev.Su(H)EnR* are brighter green. (C') Within the wild type clones there is an increase in *sev*.*lacZ* staining (red), and in the twin spots there is an enhanced reduction of expression. (C″) is a merge of (C) and (C'). The inset shows a detail from the box indicated in (C') additionally stained for *svp.lacZ* (blue) which highlights the R1/6/3/4 cells. To the right (in the wildtype tissue) R1/6 flank the R7 cell expressing *sev*.*lacZ* (red). To the left (where there are two copies of *sev.Su(H)EnR*) the cell in the R7 position (asterisk) expresses little *sev*.*lacZ* but has high levels of *svp.lacZ* (blue). (D–F) Reduction of N activity using *N^[ts]^* reduces Sev protein levels. (D) Shows a wild type disc stained for Sev (red). (E) In a *N^[ts]^* disc held at 30°C for 24 h Sev staining is very low. (F) Clones of *N^[ts]^* (labeled by the absence of GFP) held at 30°C for 24 h show an autonomous reduction in Sev expression.

To investigate this possibility we examined the expression of *sev.lacZ* in developing ommatidia of sev.N^*^ flies, and observed a rapid and strong accumulation of ectopic β-Galactosidase in the R1/6 precursors (Figure 2B), which in wild type (Figure 2A) show no detectable expression. Accordingly, we infer that N activity can up-regulate *sev* transcription in these cells, allowing the sev.N^*^ transgene to amplify its own expression as well as that of the *sev.lacZ* transgene.

To determine whether the normal up-regulation of *sev* transcription in the R7 precursor depends on N activity, we performed the reciprocal experiment of assaying *sev.lacZ* expression in ommatidia in which the N transduction pathway was impaired in two different ways. In the first approach, we used the *sev* promoter to drive expression of a chimeric protein consisting of Suppressor of Hairless (Su(H)) fused to the Engrailed repressor domain (EnR): *sev.Su(H)EnR* [20]. Su(H) can function as a transcriptional activator or repressor depending on the state of N activity [23], but Su(H)EnR is “locked” in the repressor state and acts as a constitutive repressor of N target genes. If *sev* transcription is normally up-regulated by N, the *sev.Su(H)EnR* transgene might be subject to a self-limiting, governor effect in which N activity leads to enhanced expression of Su(H)EnR, which in turn impairs the expression of Notch target genes, including both *sev* and the *sev.Su(H)EnR* transgene itself. Consistent with this reduction in N signal transduction and a requirement for N input in the R7 fate choice, R7 precursors are transformed (albeit at a low penetrance) to R1/6 fates in *sev.Su(H)EnR* flies [20].

To assess the effect of the *sev.Su(H)EnR* transgene on *sev.lacZ* expression, we generated wild type clones in a background of cells carrying a single copy of the *sev.Su(H)EnR* transgene, allowing us to compare *sev.lacZ* expression in experimental (*sev.Su(H)EnR*) versus wild type (control) cells, side-by-side. *sev.lacZ* activity was reduced cell-autonomously in R7 precursors as it was in the other cells that expressed *sev.Su(H)EnR* (R3/4 and the cone cells) (Figure 2C). Furthermore, this reduction was even more pronounced in the “twin spot” clones in which two copies of *sev.Su(H)EnR* are present (Figure 2C).

The second method we used to reduce N activity was to use the temperature sensitive allele -*N^[ts]^*. We examined eye discs that were fully mutant for *N^[ts]^* or had *N^[ts]^* clones induced in them. In these manipulations the animals were held at the restrictive temperature for 24 h before dissection and analysis. We observed a strong reduction in *sev.lacZ* expression in all cells that normally express it, in both the whole disc experiments and in the clones (not shown). Since β-Galalactocidase has a long perdurance it may give an inappropriate indication of surviving *sev* transcripts when N activity is severely reduced. We therefore monitored Sev protein levels. Here the whole disc experiments showed an almost complete loss of Sev expression (Figure 2E), and an autonomous loss was detected in the clones (Figure 2F). Again, the effects on Sev expression were universal; all cells that express Sev (including the presumptive R7) showed a loss of the protein expression.

Since raising or lowering activity of the N signal transduction pathway correspondingly raises or lowers *sev* transcription, we infer that N acts to promote high levels of *sev* expression in the R7 precursor.

#### (ii) High levels of *sev* transcription are required for R7 specification

**Figure 3:**
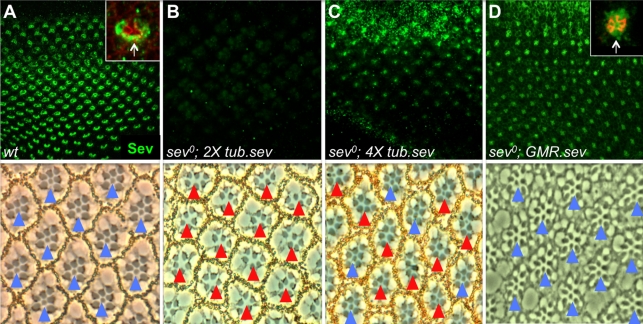
Rescue of *sev* transcription using heterologous promoters. Upper panels show the levels of Sev expression (green) in third instar eye discs, and the lower panels show the corresponding adult eyes. Blue arrows indicate ommatidia with R7s, red arrows indicate ommatidia without R7s. (A) shows a wild type eye disc stained for Sev. The inset shows a high magnification image of an ommatidial cluster counterstained with α-Arm (red) to highlight the adherens junctions. The arrow points to Sev protein in the R7 precursor. In the corresponding adult retina all ommatidia have R7 cells. (B) In *sev^0^* mutant flies carrying two copies of a *tub.sev* transgene, little Sev staining is evident in the disc, and there are no R7s in the adult retina. (C) When four *tub.sev* transgenes are introduced in a *sev^0^* fly there is more Sev expression detected in the discs, and many ommatidia show rescued R7 cells. (D) *sev^0;^ GMR.sev* eye discs show high levels of Sev expression in the eye disc. The inset shows a high magnification image of an ommatidial cluster counterstained with α-Arm (red) to highlight the adherens junctions and the arrow indicates the level of Sev protein in the R7 precursor. This level of Sev protein rescues *sev^0^* as evidenced by the presence of the R7s in the corresponding adult retina.

In the absence of the *sev* gene, the R7 precursor is inappropriately specified as a cone cell, and we next asked whether the high levels of *sev* transcription induced by N activity are indeed necessary for the appropriate R7 specification. To do this we varied the level of Sev expression independently of N signaling by replacing the endogenous *sev* gene with transgenes that express within the eye in a blanket manner. First we used the low-level, ubiquitous *tubulinα1* promoter (*tub.sev*). In *sev* null (henceforth *sev°*) flies, all R7 precursors differentiate as cone cells and the adult eyes have no R7 cells. To the *sev°* mutant background we introduced two *tub.sev* transgenes and observed no rescue of any R7s (*N* = 272 ommatidia). Correspondingly, the eye discs of this genotype showed almost no Sev expression (Figure 3B). When a different set of two *tub.sev* transgenes was tested, again no rescue of R7s was observed (*N* = 283 ommatidia). However, when the four transgenes were combined, 29% of the R7s were rescued (*N* = 344 ommatidia), and a correspondingly higher level of Sev protein was present in the eye discs (Figure 3C). GMR is a promoter element that drives at high levels in all the developing retinal cells, and into the *sev°* mutant background we introduced a *GMR-sev* transgene (*sev^o^; GMR.sev*) and observed almost complete rescue (97%) of the R7s (*N* = 511 ommatidia) with a corresponding high level of Sev expression in the eye disc (Figure 3D). Likewise, increasing the number of the *tub.sev* transgene to 8 copies leads to a complete rescue (100%, *N* = 130 ommatidia). Collectively, these data suggest that at low levels of Sev expression R7s are not specified, at moderate levels an intermediate number of R7s are rescued, and high levels correspond with a robust specification of R7s. We therefore infer that N functions to ensure a high level of Sev in the R7 precursor for its appropriate specification.

### Role 2—N Activity Specifies a Photoreceptor as the R7 Rather Than the R1/6 Type

Based on the reciprocal transformations between the R1/6 and R7 fates caused by reducing or elevating N activity, we previously proposed that N normally directs the choice of the R7 fate as an alternative to R1/6 [20]. However, we now understand that the N pathway modulates the level of *sev* transcription, and the question arises as to whether N directs the R7 versus R1/6 choice through the regulation of *sev* or through a separate mechanism.

To address this question, we used the *sev^o^; GMR.sev* flies in which there is no endogenous *sev* gene for N to up-regulate, and *sev* transcription is supplied by a heterologous promoter element that is not responsive to N. Hereafter we refer to this as the “*GMR.sev-rescued*” condition.

In this background we repeated the three genetic mosaic experiments used to define the requirement for N signaling in the R7 versus R1/6 choice [20]: namely assaying the effects of *Dl* null (henceforth *Dl ^o^*), *sev.Su(H)EnR*, and sev.N^*^ clones.

#### (i) *Dl^o^* clones in *GMR.sev-rescued* eyes

**Figure 4:**
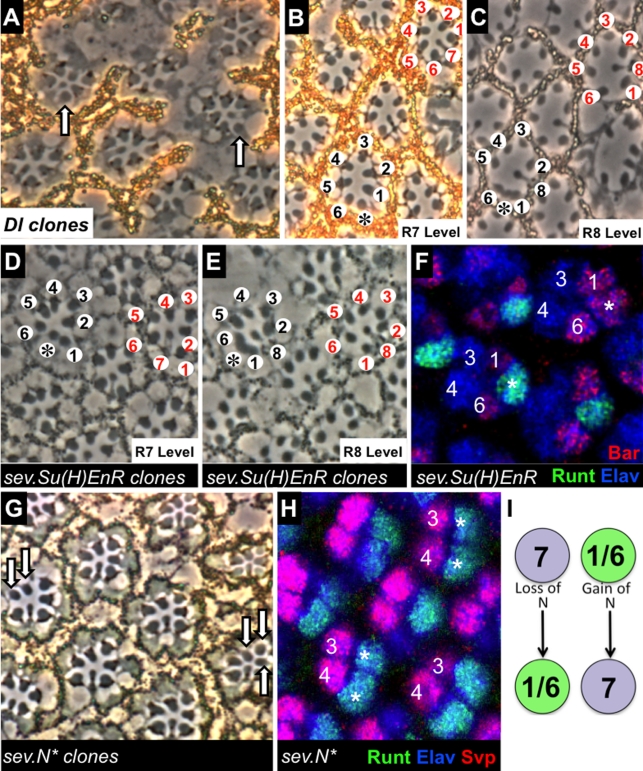
The effects of N manipulations in the *sev°; GMR.sev* background. (A–C) *Dl clones* (labeled by lack of pigment—evident in photoreceptors by the absence of the black granular mass adjacent to the rhabdomeres) in the *GMR.sev-rescued* background. (A) Mosaic analysis shows that normal ommatidia still form if either R1 or R6 is mutant for *Dl* (arrows point to R1 or R6 cells lacking Dl), but not when both are mutant. (B,C) The fate of R7 precursors when both R1 and R6 are mutant. (B) The lower ommatidium labeled with black numbers shows the cell in the R7 position (asterisk) appearing as an R1/6 type when the cells in the R1/6 positions are both *Dl*. Compare with the wild type ommatidium (top right) labeled in red. (C) At the level of the R8s, the lower ommatidium (black labels) still shows the large rhabdomere cell (asterisk) consistent with it being an R1/6 type, and R8 can be seen projecting between the inferred R1/2 cells. Compare with the wild type ommatidium (red labeling) in which the cell in the R7 position is no longer evident at this depth and R8 projects between R1 and R2. (D,E) Clones of *sev.Su(H)EnR* labeled by the absence of pigment. (D) When the cell in the R7 position carries *sev.Su(H)EnR*, it can transform into an R1/6-like cell (asterisk) with (E), a rhabdomere that projects into the R8 levels. Labeling and details are as given in (B,C) above. (F) In a *sev.Su(H)EnR* eye disc cells in the R7 position often differentiate as a normal R7 (lower left asterisk marks a Runt-expressing R7), but at a lower frequency the cell in the R7 position expresses Bar the R1/6 marker (upper right asterisk). Cells are also stained with α-Elav to mark the neural fate. (G,H) The effects of N^*^ on *sev°; GMR.sev* R1/6 cells. (G) shows a mosaic analysis of sev.N^*^ (marked by the absence of pigment). Arrows point to sev.N^*^ R2, R3, R4 and 5 cells in normally constructed ommatidia. (H) Image of a third instar sev°; GMR.sev; sev.N^*^ eye disc. The cells in the R1/6 positions express Runt (asterisks) the R7 marker. The tissue is counter-stained with α-Svp to mark R1/6/3/4 cells and α-Elav to label neurons. (I) Schematic summary of the effects of N manipulation in the *sev°; GMR.sev* background. If N signaling is reduced in the R7 precursor, it differentiates as an R1/6 type. If N signaling is activated in an R1/6 precursor, the cell is specified as an R7.

In wild type eyes, *Dl* is required in either R1 or R6 for R7 specification [20]. We performed *Dl* mosaic analysis in *GMR.sev-rescued* flies and scored normally constructed ommatidia to determine which photoreceptors could be *Dl^o^* without perturbing R7 specification. As we previously observed in wild type flies, *Dl* could be safely removed from any of the R1, R6, or R7 precursor cells (Figure 4G), provided that it was not removed simultaneously from both the R1 and R6 precursor. We scored 127 normally constructed yet genetically mosaic ommatidia (containing a mixture of wild type and *Dl^o^* cells). 30% of these had *Dl^o^* cells in the R1/6/7 group (14% had either a single R1 or R6 mutant, 15% had R1/R7 or R6/R7 mutant, and 1% had only R7 mutant). No ommatidium had both R1 and R6 mutant. Thus, correct specification of the R7 photoreceptor still requires Dl signaling from either the R1 or R6 cell, even when the requirement for N in up-regulating *sev* is bypassed.

Determining the fate of the cell in the R7 position when both R1 and R6 are mutant for *Dl* is difficult as such ommatidia are structurally aberrant, and a degree of inference is required in identification of the constituent cells [20]. Given this proviso, ommatidia were observed that had a morphologically R1/6-like cell in the R7 position when the neighboring R1 and R6 cells were both *Dl^o^* (Figure 4B and 4C), as we previously observed for *Dl* mosaics in otherwise wild type eyes [20]. Hence, in *GMR.sev-rescued* ommatidia, as in wild type ommatidia, it appears that the cell in the R7 position must receive Dl input from either the R1 or the R6 cell to avoid being mis-specified as an R1/6 cell.

#### (ii) *sev.Su(H)EnR* clones in *GMR.sev-rescued* eyes

As described above, *sev.Su(H)EnR* acts as a mild cell-autonomous suppressor of the N transduction pathway. Here, we focus on the consequences of reducing N transduction in the R7 precursor. Such analysis is complicated by the accompanying reduction of N signaling in the cone cell precursors which, as we describe below, can cause them to be mis-specified as R1/6 photoreceptors. To avoid this complication we generated mosaic eyes with only small, rare *sev.Su(H)EnR* clones, and scored ommatidia containing only one or a few mutant cells and lacking supernumerary photoreceptors. In an otherwise wild type background, a low frequency of *sev.Su(H)EnR* cells located in the R7 position differentiate inappropriately as R1/6 cells [20] and the same was observed in the *GMR.sev-rescued* background (Figure 4D and 4E).

We then examined the effects of *sev.Su(H)EnR* activity in *GMR.sev-rescued* ommatidia in the larval retina during the stage of R7 specification (but before supernumerary photoreceptors are added by mis-specification of cone cells) using molecular markers of cell fate. Here, α-Runt staining was used to label R7′s, and α-Seven-up (Svp) and α-Bar used to label the early and late phases of R1/6 specification, respectively. In an otherwise wild type background, *sev.Su(H)EnR* causes a fraction of the R7 precursors to express R1/6 markers instead of R7 markers [20], and we observe a similar mis-specification of R7 precursors in the *GMR.sev-rescued* background (Figure 4F).

Collectively, our analysis of both the *Dl^o^* and *sev.Su(H)EnR* mutant conditions argue that N activity in the R7 precursor is required to select the R7 fate instead of the R1/6 fate, even in the *GMR.sev-rescued* background, when it is no longer required to up-regulate *sev*.

#### (iii) sev.N^*^ clones in *GMR.sev-rescued* eyes

Boss is the ligand for Sev and is expressed by the developing R8 cell. Both R1 and R6 contact R8, so any Sev expressed in these cells should gain access to the ligand. And yet Boss has no effect on R1/6 specification, even when Sev is supplied at high levels in the R1/6 cells, as in *GMR.sev-rescued* ommatidia (Figure 3D), as expected if N input is required for R7 versus R1/6 specification. Similar results were found when heat shock expression of the *sev* gene was previously used to rescue *sev°* ommatidia [24],[25].

Above, we have assayed the requirement for N in promoting the R7 fate. We next examined the effects of ectopic N activity in R1/6 precursors using sev.N^*^. Our earlier studies showed that the R1/6 precursors are sensitive to the presence of ectopic N activity. Although there were some escapers, the R1/6 precursors were largely specified as R7 types [20]. We have repeated these experiments in the *GMR.sev-rescued* background and obtained the same result. Of 73 mosaic ommatidia with normal pattern only 7% of ommatidia showed R1 or R6 cells carrying the sev.N^*^ transgene. In comparison, control clones using a neutral marker showed 70% of mosaic ommatida with R1/6 cells labeled. Thus, in the *GMR.sev-rescued* background the presence of sev.N^*^ strongly interferes with the correct specification of the R1/6 fates. Furthermore, in entirely mutant sev.N^*^ GMR.sev-rescued eye discs, the vast majority of R1/6 precursors express the R7 marker, Runt, at the expense of the R1/6 marker, Svp (Figure 4H).

In summary, the level of N signaling in the R1/6 precursors, as in the R7 precursor, distinguishes between alternative R1/6 and R7 fates, even when the N-dependent requirement for *sev* up-regulation is met by other means. When a cell has been specified as a photoreceptor (Ttk degradation) the presence of high N activity dictates the R7 fate, whereas a low N signal directs the R1/6 fate (Figure 4I).

### Role 3—N Activity Imposes a Barrier to Photoreceptor Specification by DER Signaling That Can Be Overcome by Sev Signaling

The first two roles of N (up-regulating *sev* to allow the R7 precursor to initiate photoreceptor differentiation and to distinguish between the R7 and R1/6 photoreceptor fates) were gleaned primarily from examining the consequences of manipulating N activity in the R7 precursor cell. Below we present three experiments that manipulate N and Sev/Ras transduction in the R1/6 and cone cell precursors and we describe the effects on whether these cells adopt the photoreceptor (R1/6/7) or non-photoreceptor (cone cell) fate.

#### (i) In the absence of *sev*, ectopic N activity causes the R1/6 precursors to become cone cells

In both wild type and *GMR.sev-rescued* ommatidia, sev.N^*^ activity in R1/6 precursors directs them to the R7 fate. However, in both these contexts, *sev* is also up-regulated, posing the question of what fate the R1/6 precursor cells would adopt if the contribution of Sev signaling was abolished. To address this, we compared sev.N^*^ommatidia in the presence or absence of the endogenous *sev* gene.

**Figure 5:**
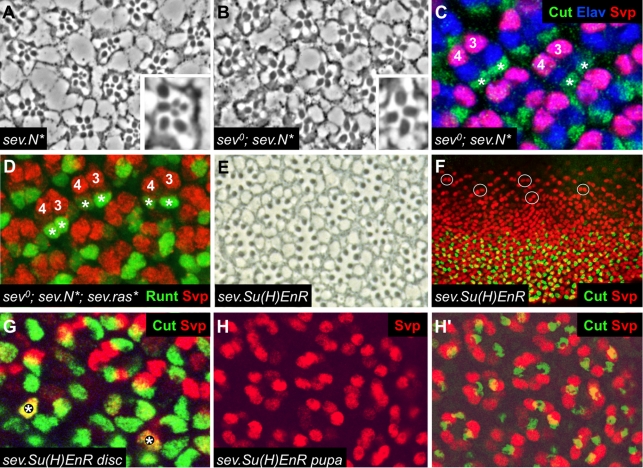
Evidence that N^*^ generates a barrier to photoreceptor differentiation. (A–D) The effects of sev.N^*^ in the absence of *sev*. (A) shows an apical section through a sev.N^*^eye; ommatidia have a variable number of photoreceptors, often four large rhabdomere cells and two or three small rhabdomere cells - see inset. (B) When *sev* is concomitantly removed (sev^0^; sev.N^*^), there is a loss of the small rhabdomere cells. (C) sev°; sev.N^*^ eye disc shows cells in the R1/6 positions (asterisks) expressing the cone cell marker Cut (green). (D) When sev.Ras^*^ is supplied to the cells shown in (C), the R7 fate is restored to the cells in the R1/6 positions (asterisks) as evidenced by Runt expression (green). (E–H) Down-regulation of N signaling converts cone cells to R1/6 type cells. (E) shows a section through a *sev.Su(H)EnR* eye; many large rhabdomere cells are present in the ommatidia. (F) A *sev.Su(H)EnR* disc labeled for Cut (green) and SVP (red). Circles highlight early R3/4 pairs showing no evidence of incorporation of mystery cells. (G) Image from the posterior of a *sev.Su(H)EnR disc* labeled as in (F). Cells in cone cell positions expressing Cut can also express Svp (asterisks). (H) Image of a *sev.Su(H)EnR* 36 h pupal disc showing supernumerary Svp-expressing photoreceptors (red). (H') The same disc as (H) with the level of Cut expression (green) flattened onto the Svp layer. There are a reduced number of Cut expressing cone cells.

Adult sev.N^*^ ommatidia (Figure 5A) are variable in structure and usually contain four large rhabdomere cells (probably corresponding to R2, R3, R4 and R5) and two to four small rhabdomere cells (probably R7 and R8 plus variable numbers of supernumerary R7 cells [26] derived from the R1 and R6 precursors [20]). The variability derives from the loss of cells from the presumptive R1/6/7 positions in sev.N^*^ ommatidial clusters. Why this happens is not known, but it results in a variable number of supernumerary R7s being generated [20]. When the *sev* gene is removed in this background, there is a dramatic reduction in the number of small rhabdomere cells, in most cases to only a single R8 cell, without a gain in the number of large rhabdomere cells (Figure 5B). From this we draw two inferences. First, the sev.N^*^-induced transformation of R1/6 precursors to the R7 fate is *sev*-dependent. Second, in the absence of *sev*, the R1/6 precursors do not default back to their normal fate since there is no corresponding increase in numbers of large rhabdomere cells.

What do the sev°; sev.N^*^ R1/6 precursors become? To address this, we stained *sev^o^*; sev.N^*^ eye discs with photoreceptor markers (Svp, Elav, Runt) and the cone cell marker (Cut) and observed that the cells in the R1/6 positions express Cut and none of the photoreceptor markers (Figure 5C). Hence, N activation in the R1/6 precursors in the absence of *sev* directs them to the cone cell fate.

#### (ii) Activated Ras restores R7s in sev^o^; sev.N^*^ommatidia

Sev is specifically required for the R7 fate, in contrast to the outer photoreceptors (including R1/6), which are specified by DER signaling. It is generally assumed that the R1/6 precursors receive Spitz, the DER ligand, from the precluster cells (most likely the abutting photoreceptors R2 and R5). Spitz binding to DER activates the Ras/MAPK pathway leading to the degradation of Ttk; the inhibitor of photoreceptor differentiation (Figure 1E; see Introduction). Thus, in normal development, activation of DER appears sufficient to promote photoreceptor differentiation in the R1/6 precursors by destroying Ttk. However, when N is inappropriately activated in these cells in the absence of sev (sev°; sev.N^*^) the cells no longer become photoreceptors, but instead become cone cells. If, however, *sev* is present (as in sev.N^*^, or sev°; GMR.sev; sev.N^*^ ommatidia), the cells differentiate as photoreceptors. Thus, N activity appears to create a barrier to photoreceptor differentiation that DER alone cannot overcome. Yet when Sev is concurrently activated the photoreceptor fate is specified. This difference could be explained in at least two ways. First, Sev and DER are different types of RTKs, and hence, Sev may be able to activate downstream transduction pathways that DER cannot. Second, Sev simply provides more of the same activity that DER provides, namely an increased activation of the Ras/MAPK pathway. Indeed, it may be that N up-regulates *sev* in the R7 precursor cells specifically to enable them to receive a more potent RTK signal than that normally mediated by DER.

To distinguish between these models, we used a sev.Ras^*^ transgene to supply high levels of constitutive Ras activity to the R1/6 cells in sev°; sev.N^*^ ommatidia, allowing us to assess whether potent activation of the canonical Ras/MAPK pathway is sufficient to overcome the sev.N^*^ induced barrier to photoreceptor differentiation. sev°; sev.N^*^; sev.Ras^*^ flies do not eclose which prevented an assessment of the adult phenotype, but in eye discs the cells in the R1/6 position develop as R7s (Figure 5D), indicating that high level activation of the Ras pathway suffices to overcome the block to photoreceptor differentiation.

#### (iii) Down-regulation of the N transduction pathway causes cone cell precursor cells to adopt the R1/6 fate

As described above, down-regulation of N signal transduction by the *sev.Su(H)EnR* transgene has at least two distinct consequences in the developing retina: first, it can cause the R7 precursor cell to adopt the R1/6 fate, and second, it can produce supernumerary large rhabdomere photoreceptors (Figure 5E). There are two likely sources of these ectopic photoreceptors: the “mystery” cells and the cone cells. Mystery cells are early companions of the R2–5 and R8 precluster cells that are subsequently lost [3], but various manipulations can induce them to differentiate as photoreceptors (e.g., [27]). To determine the source of these ectopic photoreceptors, we examined molecular markers of photoreceptors and cone cells in developing larval and pupal *sev.Su(H)EnR* discs. Nascent ommatidia in such discs show no evidence of mystery cell incorporation (Figure 5F), but at the posterior of such discs we detect defects in the cone cell array. Cell identification in the posterior tissue is difficult because *sev.Su(H)EnR* also affects the chirality of the ommatidia and they rotate in a disorganized manner compromising a simple reading of which cells belong to which ommatida. The cone cells' nuclei lie above the photoreceptor nuclei and express the transcription factor Cut, and these features make it is easier to identify the cone cell cluster of each ommatidium. We observe a reduction in the number of nuclei in the cone cell positions, and a concomitant reduction in the number of nuclei expressing Cut. Indeed, we observe nuclei in cone cell positions expressing both Cut and Svp, suggesting that such cells have a confused identity (Figure 5G). In the 36 h pupal eyes individual cells belonging to each ommatidium are easy to identify, and here we observe an increase in the number of Svp-positive photoreceptors and a decrease in the number of cone cells (Figure 5H). At this stage there is no co-expression of Svp and Cut and any previous ambiguities appear to have resolved. These data suggest that at least some presumptive cone cells transform into Svp-expressing R1/6-like cells in the *sev.Su(H)EnR* background. We note that this phenotype is only partially penetrant, likely reflecting the weak suppression of the N pathway by the *sev.Su(H)EnR* construct.

In principle, the RTK signal responsible for inducing cone cells to differentiate as photoreceptors in *sev.Su(H)EnR* mutant retinas could be Sev or by DER. A Sev signal seems unlikely as its only known ligand, Boss is restricted to R8, a cell that the cone cell precursors do not contact. To confirm this we analyzed *sev^o^*; *sev.Su(H)EnR* ommatidia and observed supernumerary photoreceptors, as in *sev.Su(H)EnR* ommatidia (not shown). Hence, we infer that DER is responsible for the mis-specification of cone cell precursors as R1/6 photoreceptors in *sev.Su(H)EnR* ommatidia.

### N activity Regulates Ttk Levels

**Figure 6:**
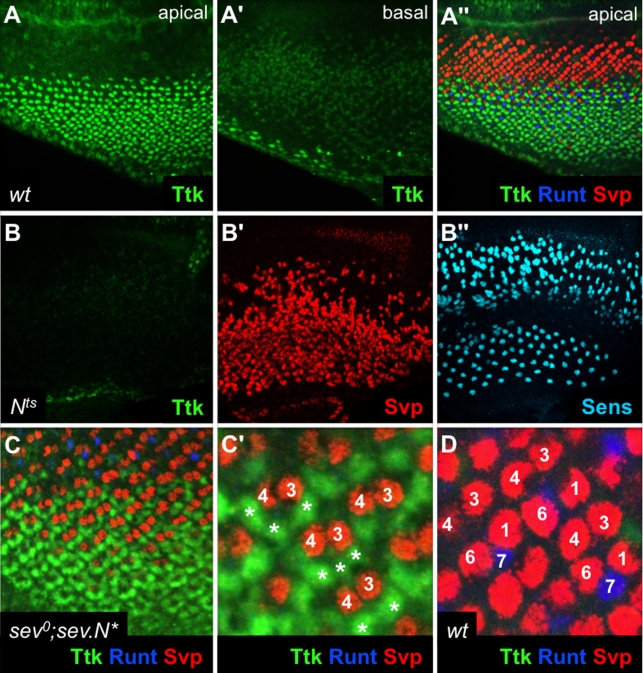
Ttk expression and its correlation with the failure to differentiate as a photoreceptor. (A) Wild type expression of Ttk. Ttk (green) is expressed at high levels in the cone cells in the apical regions. (A') But is only weakly expressed in the nuclei of the basal layer. Note, the strong staining at the back of the disc is from apical tissue curving down in the disc. (A″) shows the same disc also stained for Svp and Runt to label the R1/6/3/4 and R7/8 cells respectively, to allow clear identification of the Ttk-expressing cone cells. (B) A *N^[ts]^* disc held at 30°C for 24 h and stained for Ttk (green), which is significantly reduced. (B' and B″) show the same disc, respectively, stained for Svp (red) and Sens (blue) to show the persistent expression of other proteins. (C) A sev°; sev.N^*^ eye disc stained for Ttk (green) and Svp (red). (C') shows a blow-up in which the cells in the R1/6/7 positions (asterisks) express high levels of Ttk. (D) is a wild type disc for comparison with (C). Here R1/6/7 do not express high levels of Ttk, but instead express high levels of either Svp (R1/6) or Runt (R7).

Above we have described the evidence for a role of N in inhibiting the photoreceptor fate. We next examined the effects that N modulations have on Ttk. There are two distinct populations of cells that express Ttk in the eye disc. It is expressed at high levels in the cone cells which lie in the apical regions of the disc, and it is expressed at low levels in all the basal cells representing the cells that will subsequently be incorporated into the growing ommatidia (Figure 6A). The first three cells incorporated into the precluster are the R1/6/7 precursors, and in these cells Ttk protein is degraded. But the cone cells, which are recruited next, not only a fail to degrade Ttk but also show increased levels (compared to the basal cells from which they are derived; Figure 6A). We asked whether N activity was responsible for the high levels of Ttk in the cone cells. First, we attempted to monitor *ttk* transcriptional activity using in situ hybridization, and although we were able to detect *ttk* transcripts when the gene was over-expressed (*sev-G4; UAS-ttk*), we could not detect the wild type level of transcription (not shown), and could not therefore determine whether it was reduced when N activity was attenuated. Second, we examined the *ttk-lacZ* reporter line [7] and did not detect any down-regulation in the *N^[ts]^* background (not shown).

We next asked what happens to Ttk protein levels when N activity is reduced. Figure 6B shows protein expression in a *N^[ts]^* eye disc that has been held at the restrictive temperature for 24 h. Control stainings for Svp and Senseless show robust expression levels and pattern disruptions typical of loss of *N* function. For example, Senseless is expressed in the R8 cells, and multiple Senseless-expressing cells are induced in the anterior regions of such discs (Figure 6B″), consistent with a large depletion in N activity. In these discs Ttk accumulation is severely reduced (Figure 6B). This observation allows us to correlate the loss of Ttk expression with the loss on N activity, and we infer that when the block on photoreceptor determination is removed there is a corresponding loss of Ttk protein. We next examined the Ttk protein levels in the R1/6/7 cells of sev^0^; sev.N^*^ eye discs. These cells are normally destined to form photoreceptors, but the presence of the high-level N activity instead specifies them as cone cells. In these cells we see ectopic high levels of Ttk protein (Figure 6C), and the effect of N in blocking the photoreceptor fate correlates with ectopic high levels of Ttk protein. Thus when N signaling is raised or lowered there is a corresponding raising or lowering of Ttk levels that mirrors the effects of N in blocking photoreceptor specification.

## Discussion

We examine here the signaling that specifies three Drosophila ommatidial cell types: the R1/6 and R7 photoreceptors and the non-neural cone cells. We separate the fate choice into two binary decisions. The first is RTK-dependent and determines whether a cell is specified as a photoreceptor or not. If RTK signaling is sufficiently high, Ttk is degraded and the cell becomes a photoreceptor (Figure 1E); if not it becomes a cone cell. The second binary decision occurs once the choice to become a photoreceptor is made and dictates either the R7 or R1/6 fate. Here we examine the function of N in these two binary decisions and infer that it plays at least three roles. Two of these relate to the decision to become a photoreceptor: one role promotes the photoreceptor fate and the other opposes it. The third role distinguishes between the R7 and R1/6 types. We infer that all three roles operate in the specification of R7 itself, and such a complexity of N signaling was hitherto unsuspected. Below we evaluate the evidence for these three roles.

### 

#### (i) N activates *sev* transcription to allow R7 precursors to adopt the photoreceptor fate

Three pieces of evidence indicate that N signaling regulates *sev* expression in the R1/6, R7 and cone cell precursors. First, ectopic N activity cell-autonomously up-regulates *sev* transcription. Second, and conversely, compromising N activity reduces *sev* transcription. Third, both the sev.N^*^ and *sev.Su(H)EnR* transgenes, which depend on the N-responsive *sev* enhancer, show the expected auto-regulatory behavior: sev.N^*^ is a potent amplifier of its own expression, whereas *sev.Su(H)EnR* is subject to a “governor effect” which limits its own expression.

Furthermore, we provide evidence that the high levels of *sev* supplied by N activity are essential for R7 specification. When *sev* is low or absent R7 precursors differentiate as cone cells, but when *sev* levels are high they differentiate as photoreceptors.

#### (ii) N activity dictates the choice of the R7 versus the R1/6 fate independently of the requirement for N in up-regulating *sev*

In the *GMR.sev-rescued* background, *sev* transcription is supplied at high levels in a blanket manner. Importantly, the endogenous *sev* gene is absent and cannot be up-regulated by N signaling. In this background we have manipulated N signaling in three different ways, and in all cases we find that high N activity dictates the R7 fate whereas low activity dictates the R1/6 fate. Thus, independent of its role in specifying photoreceptor versus non-photoreceptor (up-regulation of *sev* transcription), N activity determines the type of photoreceptor that an R1/6/7 precursor becomes.

#### (iii) N activity creates a barrier to photoreceptor differentiation

N has long been implicated in antagonizing photoreceptor specification. For example, when N function is reduced in developing eyes in the region of the furrow, almost all cells become specified as photoreceptors [28]. Our work here has defined a specific inhibitory role for N within the R7 precursor.

**Figure 7:**
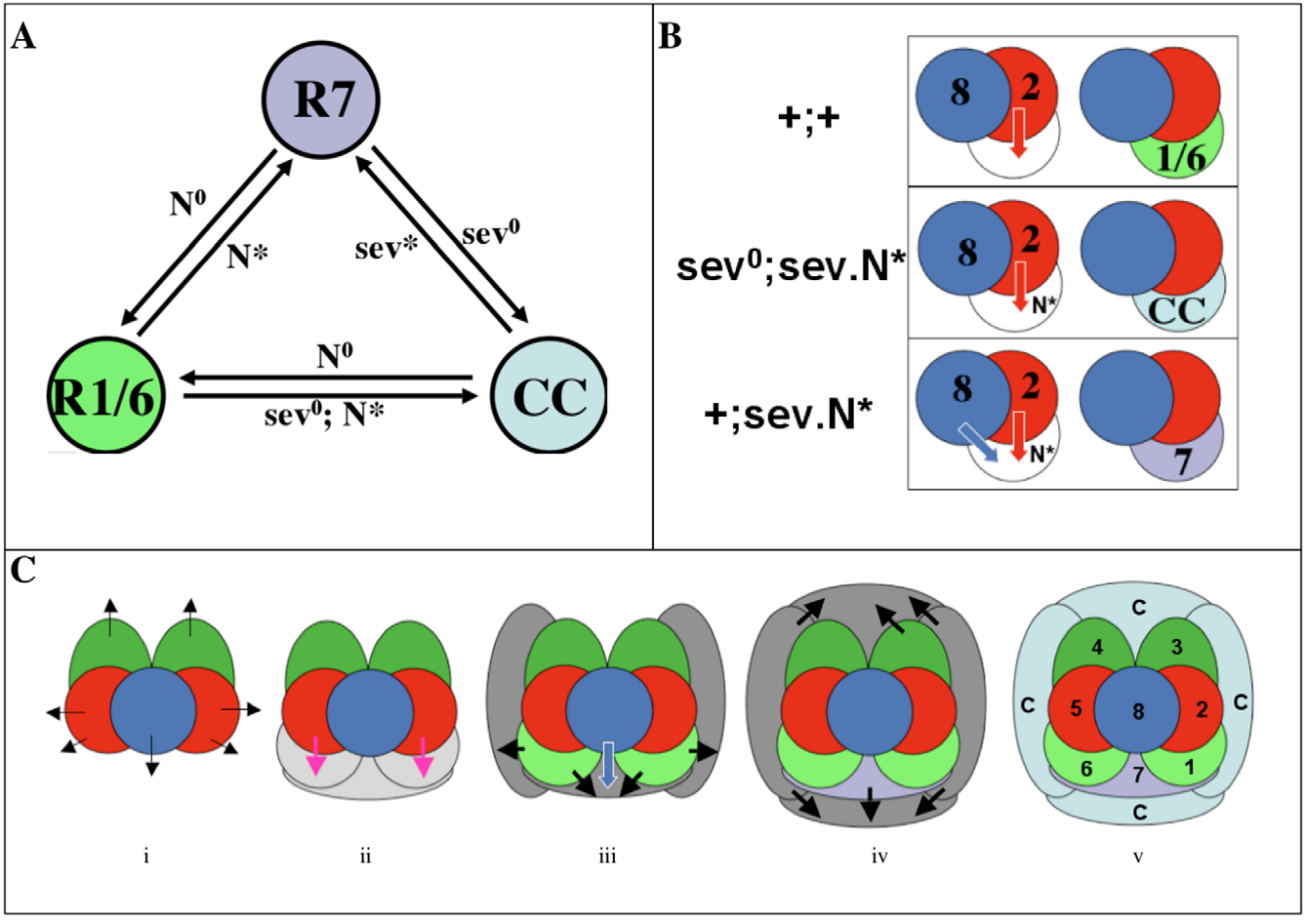
Summary diagrams. (A) R7, R1/6 and the cone cells form an equivalence group. Manipulations of the RTK/N pathways can freely transform fates between the R7, R1/6 and the cone cell types. Any cell can form any of the three fates depending on the signals that it receives. (B) N activates a repressor of photoreceptor development that DER cannot overcome but Sev can. In the upper level, the wild type situation is depicted. Here we infer that a DER activating signal from the R2 (red arrow) specifies the R1 precursor as a photoreceptor (R1/6 type). In the middle level, when N is ectopically active in the R1 precursor in the absence of sev, the DER activating signal is unable to specify the R1 precursor as a photoreceptor. In the lower level, when N is ectopically active in the R1 precursor in the presence of the *sev* gene, the cell is specified as a photoreceptor (R7 type). (C) A model of fate R1/6/7/cone cell specification. (i) The precluster cells express low levels of *Dl* (black arrows). (ii) Cells are recruited into defined niches; the first three being those on the R5/8/2 face. Three cells occupy these positions and Dl from the precluster activates mild N signaling (weak shade of gray), which provides a weak block to photoreceptor specification. Spitz expressed by R2/5 (red arrows) activates DER in the cells in the R1/6 position and overcomes the N block. (C) R1/6 begin differentiation as photoreceptors and express high levels of Dl as cells join the niches of the two flanking cone cells. The cell in the R7 position and these presumptive cone cells receive Dl from R1/6 and N is activated to high levels (dark gray). This provides a potent barrier to photoreceptor specification and also activates *sev* transcription. Binding of Sev to Boss on R8 provides high-level RTK activity (blue arrow). (iv) The RTK signaling specifies the R7 precursor as photoreceptor and the concomitant presence of activated N directs the R7 rather than the R1/6 photoreceptor type. R7 proceeds to express high levels of Dl, as do the two cone cells and R3. As the subsequent cone cells join, they receive these Dl signals and activate both the barrier to photoreceptor differentiation and *sev* expression. (V) None of the cone cell precursors contact R8 so none have activated Sev, and Spitz diffusing from R2/5 is unable to trigger the photoreceptor fate because of the high N activity.

In wild type ommatidia DER signaling directs the R1/6 precursor cells to adopt the R1/6 fate. However, if N is ectopically activated in these cells and *sev* is concomitantly removed (*sev^o^*; sev.N^*^) they become cone cells. In contrast, if we leave *sev* function intact (sev.N^*^) the R1/6 precursors develop as R7 photoreceptors. Hence, we infer that N activity imposes a barrier to photoreceptor specification that cannot be overcome by DER signaling but can be negated when Sev is active (Figure 7B). Notably, we can also induce these cells to differentiate as R7 photoreceptors if we supply them with constitutively active Ras rather than native Sev function.

Since high-level Ras activation overcomes the N-induced block, we infer that Sev is able to activate Ras to a higher level than DER. Although DER appears insufficient to overcome the block, we have not yet activated Sev in the absence of DER to determine whether it alone can overcome the inhibition.

The cells in which the block is active also have high levels of Ttk expression. From this we surmise that N activity works indirectly to prevent the degradation of Ttk. We expect this block to act somewhere between the RTKs themselves and the final output of Ttk degradation (e.g., at any of the steps and their intermediates shown in Figure 1E).

### A Model of Cell Fate Specification in the Developing Drosophila Eye

N signaling in the presumptive R7 appears to activate two competing pathways: one repressing the photoreceptor fate and the other facilitating it. Why would this be? We speculate that it relates to an ancient function of N in limiting photoreceptor number. The *phyll* mutation was named [5] after Phyllopoda crustaceans in which the ommatidia have only five photoreceptors [29], corresponding to the R2, R3, R4, R5 and R8 precluster cells. We suggest that in the ancient condition the five photoreceptor cells express Dl (or another N ligand) to prevent additional cells from becoming photoreceptors. With this view in mind we propose the following model of cell fate assignments for the cells that join the precluster (Figure 7C).

(i) The precluster cells express a moderate level of Dl.

(ii) Three cells join the ommatidium in the R1/6/7 positions, receive the Dl signal and experience mild N activation. Those in the R1/6 positions contact R2 or R5 and occlude the cell in the R7 position from doing so. R2/5 release Spitz, which activates DER in the neighboring R1/6 precursors. Since N signaling is mild in these cells the DER activation suffices to specify the cells as photoreceptors. Spitz is a diffusible ligand and will later activate DER in the cell in the R7 position.

(iii) Dl is expressed by all cells of the ommatidia as they begin to differentiate [30],[31] and R1/6/7 appear to express higher levels than the precluster photoreceptors [32]. Hence as R1/6 begin to differentiate they induce a high-level of N activation in the R7 precursor and induce a N activity level in the flanking anterior and posterior cone cells, which is too high to be overcome by the DER activation, triggered by the arriving Spitz molecules. The N signaling also turns on *sev* transcription, providing high levels of Sev to these cells. R7 contacts the Boss-expressing R8 cell leading to a high-level activation of the Ras/MAPK pathway that overcomes the N barrier and allows photoreceptor specification. The presence of activated N then specifies the cell as an R7 rather than an R1/6 type.

(iv) As R7 and the cone cells begin differentiating they express Dl. On the other side of the cluster the R3/4 pair undergo a N/Dl interaction leading to high Dl expression in R3. These Dl expressions then activate N in the two further cone cells that join the ommatidium. The four cone cells experience high N activity and express high levels of Sev, which remains inactive because the cells do not contact R8. With Sev signaling inactive, the high-level N activity suffices to prevent the diffusing Spitz signal from specifying these cells as photoreceptors.

### The Roles of DER and the Diffusion of Its Ligand

Implicit in the model above is the idea that Spitz diffuses from the precluster and reaches more distant cells with time. This concept was introduced by Freeman [14] and it explains many of the results we observe. First, consider the R7 precursor in which N activity is reduced. This cell becomes an R1/6 type. We suggest that when diffusing Spitz reaches this R7 precursor it finds the N-induced barrier absent and DER activation suffices for photoreceptor specification. Second, consider the cone cell precursors in which N signaling is also reduced. Here again the photoreceptor inhibition is weakened, and when the diffusing Spitz reaches them they too are specified as photoreceptors. Thus, we infer that in the absence of the strong N activity (providing a barrier to photoreceptor specification), diffusing Spitz can liberally induce the formation of ectopic photoreceptors.

### The RTK and N Signals Propagate by Different Means

Above we consider the case of the cone cell which receives high N activation from Dl expressed by its differentiating neighbors, and DER activation from Spitz diffusing from the R2/5 cells. Here we see two antagonistic signals propagating from the precluster and reaching distant cells by two distinct mechanisms. The N signal is relayed by the sequential incorporation of cells into the growing structure; as they begin to differentiate they express Dl. By this cell-to-cell propagation, the Dl signal progressively reaches more distant cells. The DER activation occurs by the diffusion of Spitz; it reaches more distant cells with time.

### The Niche Model of Cell Incorporation

A key concept in the model presented here is that cells are “blind” to the fate-specifying signals emanating from the precluster until they enter the ommatidium. That is, cells undergo a two-step process of cell fate specification. First they occupy a niche (a specific position into which a cell can be recruited) and then they receive their fate-specifying signals. Consider the cluster shown in Figure 1F. In image (i) the precluster is surrounded by many cells. From these, three are recruited to the R1/6/7 positions (white cells – image (ii)). We suggest that these cells now respond to the signals from the cluster, the others do not. As more cells differentiate more niches become available and more cells become incorporated. The molecular nature of this recruitment mechanism remains unknown, but it plays a critical role in preventing the ectopic and premature responses of the cells to the RTK and N signaling pathways.

### R1/6/7 and the Cone Cells Form an Equivalence Group

Since *sev^0^* R7 cells became cone cells, and cone cells with activated Sev became R7s, these cells were thought to belong to an equivalence group [27]. How this R7/cone cell equivalence group was established separate from the other cells that join the precluster remained unclear. Here by manipulation of N and RTK signaling in the R1/6, R7 and cone cells, we could transform any one of these into any other (Figure 7A). We infer therefore that all these cells form an equivalence group. Before they enter the ommatidium they are equipotent and it is the signals provided by the growing cluster that directs their fate rather than any pre-pattern that may exist in these cells prior to their incorporation.

### DER Signaling Independent of Photoreceptor Specification

We have highlighted here the role of RTK signaling in the specification of photoreceptor fate, but we note that it is also required for the appropriate specification of the cone cells [12],[14]. These cells receive a high N signal (and the concomitant barrier to photoreceptor specification) but appear to adequately transduce DER for the role in the cone cells. This again raises the question of where in the RTK pathway the N-induced barrier acts. The RTK pathway can be separated into the canonical transduction from the membrane to the nucleus, and the specific gene targets in the nucleus. Consider the photoreceptor pathway, here *phyll* transcription is required for the degradation of Ttk, and anything lowering *phyll* transcription or its downstream outputs would oppose photoreceptor specification but leave other RTK outputs unaffected. Here we view the barrier as specifically targeting the photoreceptor output of the RTK pathway. Conversely, the barrier could affect the transduction through the cytoplasm to the nucleus and affect all outputs. In this situation we envisage that cone cell specification requires only a low-level activation of the RTK pathway.

### Conclusions

We have examined what initially appeared to be a relatively simple process in cell fate assignment: the mechanism by which RTK and N signaling specify the R7 fate. We uncovered a complex signaling inter-relationship that probably results from the evolutionary history of the R7 cell. What appears paradoxical from the developmental perspective may be expected from the evolutionary view. The tractability of R7 as a model for fate specification has allowed the many functions of N to be uncovered here, but in other studies where manipulations cannot be as effectively performed, such complexity may go unrecognized.

## Materials and Methods

All methods were previously described [20], except for PLP fixation, which was used for the Ttk antibody following [3].

### Stocks

*sev[d2], Dl[rev10], sev>w+>N[act], sev>w+>Su(H-EnR*), *sev.N[ecn]* [26], *sev.lacZ* [33], *Ttk.lacZ* [7], N^[ts]^. To make *sev.Su(H)EnR* clones the following chromosome was constructed: *hs-flip, ubi-GFP, sev.Su(H)EnR, FRT 19A*. This was crossed to *FRT 19A* and heat shocked for 1 h at 37°C between 24 and 48 h AEL.

### Antibodies

Elav.7E8A10, Cut (DSHB), Runt (gift J. Reinitz), Svp (gift Y. Hiromi), Bar (gift K. Saigo), Ttk (gift P. Badenhorst), GFP (Molecular Probes), Senseless (gift H. Bellen), Sev (Santa Cruz Biotechnology), and Beta-Galactocidase (Cappel).

### *N^[ts]^* Experiments

*N^[ts]^* flies were reared at 18°C and shifted to 30°C in the third instar for 24 h followed by dissection. For clones, *N^[ts]^* was recombined with *FRT 19A* and clones were induced with *hs-flipase* or *eye-flipase*.

## Abbreviations

Boss: Bride of Sevenless
DER: Drosophila EGF-Receptor
EnR: Engrailed repressor domain
N: Notch
*phyll*: *phyllopod*
RTKs: receptor tyrosine kinases
Sev: Sevenlesss
Su(H): Suppressor of Hairless
Svp: Seven-up
Ttk: Tramtrack
